# How much does a Medical and Healthcare Products Regulatory Agency medical device alert for metal-on-metal hip arthroplasty patients really cost?

**DOI:** 10.1177/1120700020983297

**Published:** 2021-01-14

**Authors:** Rajpal S Nandra, Usman Ahmed, Fiona Berryman, Lesley Brash, David J Dunlop, Gulraj S Matharu

**Affiliations:** 1 The Royal Orthopaedic Hospital, Birmingham, UK; 2 Musculoskeletal Research Unit, Translational Health Sciences, Bristol Medical School, Southmead Hospital, Bristol, UK

**Keywords:** Cost, hip arthroplasty, metal-on-metal, revision surgery, surveillance

## Abstract

**Background:**

Many worldwide regulatory authorities recommend regular surveillance of metal-on-metal hip arthroplasty patients given high failure rates. However, concerns have been raised about whether such regular surveillance, which includes asymptomatic patients, is evidence-based and cost-effective. We determined: (1) the cost of implementing the 2015 MHRA surveillance in “at-risk” Birmingham Hip Resurfacing (BHR) patients; and (2) how many asymptomatic hips with adverse reactions to metal debris (ARMD) would have been missed without patient recall.

**Methods:**

All BHR patients eligible for the 2015 MHRA recall (all females, and males with head sizes ⩽46 mm, regardless of symptoms) at one centre were invited for review (hips = 707; patients = 593). All patients were investigated (Oxford Hip Score, radiographs, blood metal ions, and targeted cross-sectional imaging) and managed accordingly. Surveillance costs were calculated using finance department data.

**Results:**

The surveillance cost £105,921.79 (range £147.76–£257.50/patient). Radiographs (£39,598) and nurse practitioner time/assistance (£23,618) accounted for 60% of overall costs. 31 hips had ARMD on imaging (12 revised; 19 under surveillance). All revisions were symptomatic. 7 hips with ARMD under surveillance were asymptomatic and remain under regular review. The number needed to treat to avoid missing one asymptomatic ARMD case was 101 patients, representing a cost of £18,041 to avoid one asymptomatic case.

**Conclusions:**

Implementing MHRA surveillance for “at-risk” BHR patients was extremely costly. The risk of asymptomatic ARMD was low with the BHR (1%), suggesting recommended follow-up in asymptomatic patients is not cost efficient. This raises concerns about the increasingly intensive surveillance recommended in the 2017 MHRA guidance for metal-on-metal hip patients.

## Introduction

Metal-on-metal hip arthroplasty (MoMHA), in the form of stemmed total hip replacement and hip resurfacing, has experienced high implant failure rates.^1,2^ Many revisions have been performed for adverse reactions to metal debris (ARMD), which may develop in asymptomatic patients.^3,4^ Since 2012 worldwide regulatory authorities (including the UK, USA, Europe, Australia, and Canada) recommend regular follow-up of patients with these devices so problems can be identified and treated early.^5–9^ Surveillance can include blood metal ions, radiographs, and cross-sectional imaging, although the recommendations given by each regulatory authority are variable given the lack of evidence.^10^

In June 2017 the Medical and Healthcare Products Regulatory Agency (MHRA) in the UK updated its previous follow-up advice for MoMHA patients.^11^ This follow-up was more intensive than previous recommendations with most patients, even those with no symptoms, now needing annual investigation for the lifetime of the implant. However the clinical evidence to support the more intensive surveillance is questionable, and it has been suggested that the substantial increase in follow-up costs are unlikely to be offset by detecting the suspected small proportion of asymptomatic patients with ARMD who would otherwise be missed.^12,13^ Despite MoMHA only being used very rarely now,^14^ the burden of this problem for both patients and healthcare systems will continue for many years.^13^ Although some have attempted to estimate the financial costs of implementing such a recall, these have been based on national data and do not consider specific costs (including staffing clinics and administration) and the subsequent results of each investigation.^10^

In June 2015 the MHRA released a Medical Device Alert (MDA) involving all female patients with Birmingham Hip Resurfacings (BHRs: Smith & Nephew, Warwick, UK) and all male patients with BHR head sizes 46 mm or below, regardless of symptoms. The recommendations were that patients should have an annual review with investigations including blood metal ions and cross-sectional imaging.^15^ This was a substantial increase in the surveillance burden compared with the earlier 2012 recommendations, where these patients could be followed up according to local protocols, which in some cases meant patients were discharged or remotely followed up.^5^ After the 2015 MHRA MDA^15^ was published our institution recalled all patients with BHRs considered “at-risk” of developing problems for surveillance.

We determined: (1) the institutional cost of implementing the 2015 MHRA surveillance in “at-risk” BHR patients; and (2) how many asymptomatic hips with evidence of ARMD would have been missed if patients were not recalled. Addressing these questions would importantly allow us to establish whether or not the cost of widespread surveillance was offset by the early detection of asymptomatic patients with ARMD who potentially needed revision surgery.

## Methods

We performed a prospective cohort study of all BHR patients subject to the June 2015 MHRA recall. This included all female patients (regardless of symptoms), and all male patients with head sizes ⩽46 mm (regardless of symptoms).^15^ This study had institutional approval, and was conducted as per national guidance.

Patients requiring recall were identified from our prospectively maintained clinical database between June and December 2015. Any patients already under active surveillance at our centre (i.e. had scheduled clinic appointments within the next 1 year) were not formally recalled. The remaining patients, regardless of symptoms, were all asked to complete and return an Oxford Hip Score (OHS) postal questionnaire (patients with bilateral hips were sent scores for each hip), which is a validated patient-reported outcome measure.^16^ The OHS was used to make an assessment of whether or not patients were symptomatic, and an OHS below 27 out of 48 was considered suggestive of symptoms as detailed previously.^17,18^ Upon receipt of the completed questionnaire all patients, regardless of symptoms, were then invited by postal letter to attend dedicated arthroplasty follow-up clinics for evaluation. A second clinic appointment was offered if patients did not attend the initial review. For the patients who did not attend either of the initial 2 clinic appointments offered, postal letters were sent to the general practitioner and the patient requesting they make contact with the hospital to arrange a convenient appointment.

Clinics were arranged to swiftly see all patients from the 2015 recall predominantly within the existing organisational framework, as patients with stemmed MoMHAs from our centre were already being reviewed in a similar manner in dedicated clinics since 2012.^5,19^ Additional weekend clinics were also arranged to facilitate review of BHR patients identified from the 2015 MHRA surveillance. 2 clinic sessions per week (each 4 hours) were used for the BHR recall. In addition, 1 extra weekend clinic per month (8 hours) was also used for the recall. The 2 midweek clinics could see a maximum of 38 patients per week, and the weekend clinic could see a maximum of 38 patients (approximately 152 patients per month if all appointments used).

In the clinic, all patients were reviewed by an Advanced Nurse Practitioner experienced in assessing and managing hip arthroplasty patients. Practitioners were supported by healthcare support workers and phlebotomists. Each appointment included a 15-minute consultation (history and clinical examination, including gait), radiographs, and blood metal ion sampling. Patients had anteroposterior pelvic radiographs, with or without lateral hip radiographs, which were assessed in detail by consultant surgeons for abnormalities as described.^20^ Whole blood cobalt and chromium concentrations were measured at an MHRA accredited laboratory, with methods described previously.^21^

The 2015 MHRA recall recommended cross-sectional imaging should be performed in all “at-risk” BHR patients, regardless of symptoms (although this has subsequently been downgraded to targeted cross-sectional imaging in asymptomatic hip resurfacing patients in the 2017 MHRA recommendations which are currently used in our country).^15^ However, at the time of the 2015 recall, our institution considered the substantial cost and resource implications of the recall given the high number (over 5000 MoMHAs, which included all patient groups and a number of different implants) of MoMHAs implanted at our centre, and the good results we achieved with the BHR.^22,23^ Therefore a selective policy was agreed locally for performing cross-sectional imaging in recalled patients. Any patient exhibiting 1 or more of the following features underwent cross-sectional imaging: (1) symptoms, and/or limping, noises from the hip, instability, abnormal clinical examination; (2) cobalt and/or chromium concentrations above the 7 μg/l MHRA threshold; and/or (3) abnormality on radiographs (including component malposition, loosening, neck thinning).^5,24^ The institution’s protocol was metal artifact reduction sequence (MARS) magnetic resonance imaging (MRI) and/or ultrasound as described.^21^ The techniques used for each cross-sectional imaging modality at our institution have been detailed previously.^25,26^

The advanced nurse practitioners had 7.5 hours per week of administrative duties for recalled BHR patients. This included identifying appropriate patients for surveillance, organising appointments, managing any patient correspondence, reviewing the outcome of investigations (OHS, radiographs, blood metal ions, and cross-sectional imaging), and maintaining clinical records. Patients with abnormal investigations were discussed with one of the consultant hip surgeons, with further consultations, investigations, and surgery organised as required.

### Statistical analysis

To describe continuous variables with normal distributions we used the mean and range, with the median and interquartile range (IQR) used for non-normally distributed variables. Categorical variables were summarised with frequencies and percentages.

The cost for each aspect of the MHRA surveillance was obtained from our finance department (Table 1). These costs broadly included staff salary, clinics, administration, and investigations. The total institutional cost of the MHRA surveillance to review and investigate all eligible patients once was calculated by summation of the costs of the various aspects described. Using the number of patients with ARMD (on imaging and/or requiring revision) who were asymptomatic, we calculated the number needed to treat to avoid missing 1 case of asymptomatic ARMD by introducing the recommended MHRA surveillance.

**Table 1.** Summary of institutional costs for each aspect of follow-up.

Item	Price	Details
Clinic overheads	£70	36 clinics × 70 = £2520
Staff (per hour) – Weekday^*^		
– Advanced Nurse Practitioner	£35.44	2 per week–clinic (8 hours)
		4 per week–admin (15 hours)
– Support worker Band 2	£13.78	2 per week–clinic (8 hours)
		2 per week–admin (15 hours)
– Phlebotomist	£14.18	2 per week–clinic (8 hours)
– Admin support Band 4	£16.34	1 per week–admin (7.5 hours)
Staff (per hour) – Weekend^*^		
– Advanced Nurse Practitioner	£47.12	2 per month–clinic (5.5 hours)
– Support worker Band 2	£19.72	1 per month–clinic (5.5 hours)
– Phlebotomist	£19.29	1 per month–clinic (5.5 hours)
Blood Tests		
– Metal ions (cobalt and chromium)	£25	Per patient
Radiology		
– X-ray × 1	£30.46	1 hip × 2, 2 hips × 3
– USS (per hip)	£30.79	
– MARS MRI Pelvis (both hips)	£79.26	
Stationery		
– Envelopes (each)	£0.10	2 per patient
– Printing (per side A4)	£0.01	OHS 2 sides, Invitation 1 side
– Postage	£0.58	2 per patient

USS, ultrasound scan; MARS MRI, metal artifact reduction sequence magnetic resonance imaging.

* including 18% mark-up for staff overheads.

## Results

### Patient recall

**Figure 1.**
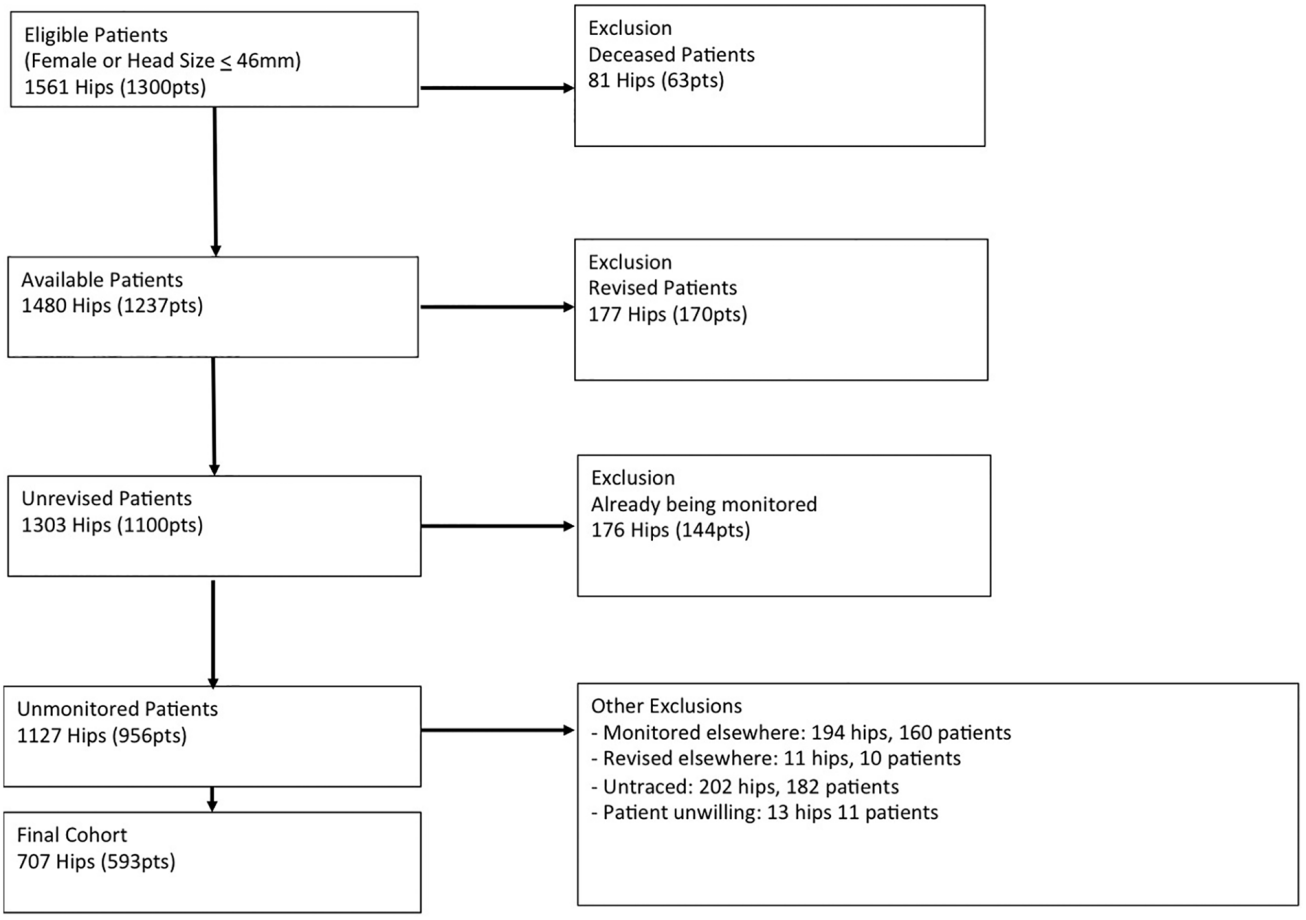
Study selection criteria.

There were 1561 hips eligible for inclusion. After excluding patients who died (*n* = 81), were revised at our centre (*n* = 177), and who were already under surveillance (*n* = 176) there were 1127 hips requiring recall for surveillance given the MHRA guidance (Figure 1). Of these cases, further exclusions were made: patients monitored elsewhere (*n* = 194), patients revised elsewhere (*n* = 11), patients declined any monitoring (*n* = 13), and patients who were untraceable despite multiple attempts at contact (*n* = 202).

There were 707 hips in 593 patients who were assessed and investigated for the 2015 MHRA recall (Table 2). All operations, performed before the recall date, were carried out by 12 different consultant arthroplasty surgeons. 1 surgeon performed just over half (*n* = 358), and of the remaining 11 surgeons the median number of BHRs performed was 22 (range 1–89).

**Table 2.** Patient demographics.

	Number (%) unless otherwise stated
**Female/Males**	524 (88.4%)/69 (11.6%)
**Mean age (range) in years**	50.7 (14.0–76.0)
**Primary diagnosis**	
Primary osteoarthritis	565 (79.9%)
Dysplasia	68 (9.6%)
Other	74 (10.4%)
**Femoral head size (in mm)**	
38	7 (1.0%)
42	142 (20.1%)
44	7 (1.0%)
46	443 (62.7%)
48	14 (2.0%)
50	86 (12.2%)
54	7 (1.0%)
58	1 (0.14%)
**Mean follow-up time since primary (range) in years**	12.0 (0.6–20.4)

### Investigations

The mean follow-up for patients following index BHR surgery was 12 years. All 593 patients provided an OHS, regardless of symptoms, and all had blood metal ions. The median OHS was 46 (IQR 37–48). The median cobalt and chromium concentrations were 2.32 μg/l (IQR 1.51– 3.80 μg/l) and 1.65 μg/l (IQR 0.94–2.89 μg/l) respectively, with 8.9% (*n* = 53) of patients having blood cobalt and/or chromium concentrations above 7 μg/l.

Cross-sectional imaging was performed in 281 patients (103 MRI, 137 unilateral ultrasound, 41 bilateral ultrasound). There was imaging evidence of ARMD in 31 hips (30 patients). Of these cases, 12 hips (12 patients, of which 11 were female) were revised for histopathologically confirmed ARMD (with or without raised metal ions) and all of these 12 hips were symptomatic (Table 3). The remaining 19 hips (18 patients) with ARMD on imaging remain under regular clinical surveillance with repeat ions and imaging. In all of these cases, imaging demonstrated small thin-walled cystic lesions with no adverse features, including no soft-tissue abnormalities (atrophy, destruction, invasion into other tissue planes) and no osteolysis or component loosening. Of these 19 hips, 7 were asymptomatic (1% of cohort screened had asymptomatic ARMD). From the cohort under surveillance there was 1 further revision for symptomatic aseptic femoral component loosening (Table 3). This patient had normal blood metal ions and imaging, with no intraoperative or histopathological evidence of ARMD.

**Table 3.** Patients under surveillance subsequently undergoing revision surgery.

	Age at primary/sex	Femoral head size (mm)	Cobalt (μg/l)	Chromium (μg/l)	Indication for primary	Indication for revision (all hips were symptomatic)	Time implant *in situ* (years)
1	74 M^*^	42	6.08	8.79	Osteoarthritis	ARMD	16.0
2	70 F	46	3.12	4.60	Osteoarthritis	ARMD	11.6
3	58 F	50	26.47	35.22	Dysplasia	ARMD	10.4
4	51 F	46	12.22	19.59	Osteoarthritis	ARMD	10.1
5	67 F	50	2.65	1.36	Osteoarthritis	ARMD + loose cup	11.0
6	74 F	42	4.68	11.33	Osteoarthritis	ARMD	14.0
7	42 F	46	1.35	0.29	Osteoarthritis	Loose stem	16.8
8	76 F	46	4.99	17.93	Osteoarthritis	ARMD + lysis stem	15.0
9	62 F	50	11.49	33.86	Osteoarthritis	ARMD	11.9
10	73 F^*^	46	17.21	23.3	Osteoarthritis	ARMD	19.0
11	61 F	42	21.58	29.14	Osteoarthritis	ARMD	16.6
12	59 F	46	26.67	33.57	Osteoarthritis	ARMD	17.8
13	75 F^*^	46	7.85	9.73	Inflammatory arthropathy	ARMD	20.8

ARMD, adverse reactions to metal debris; F, female; M, male; OHS, Oxford Hip Score.

* Patients with bilateral Birmingham Hip Resurfacings, but only 1 hip revised.

### Cost of surveillance

The total institutional cost of the MHRA surveillance to review and investigate all eligible patients was £105,921.79 (mean £178.62 per patient: range £147.76–£257.50 per patient) (Table 4). The most expensive aspects of surveillance were radiographs (£39,598), advanced nurse practitioner assistance (clinics and administration: £23,618), cross-sectional imaging (£14,828), and blood metal ions (£14,825). If all patients had undergone cross-sectional imaging the estimated cost for this particular aspect would have risen to £31,292.

**Table 4.** Cost of patient surveillance.

Item	Total cost
Clinic overheads	£2520
Clinic staff (total)	
– Advanced Nurse Practitioner	£6608.90
– Support worker/Phlebotomist	£4437.89
Blood Tests	
– Metal ions	£14,825.00
Radiology	
– X-rays	£39,598.00
– USS	£6743.01
– MRI pelvis	£8084.52
Stationery	
– Envelopes, Printing, and Postage	£826.55
Administration (total)	
– Advanced Nurse Practitioner	£17,008.99
– Administration support	£5268.94
**TOTAL SPEND =**	£105,921.79

USS, ultrasound scan; MRI, magnetic resonance imaging.

### Number needed to treat

The number needed to treat (NNT) to avoid missing 1 case of asymptomatic ARMD requiring revision surgery could not be calculated, as all 12 patients identified from screening with ARMD who needed revision were symptomatic. The NNT to avoid missing 1 case of asymptomatic ARMD on imaging and not requiring revision by introducing the recommended MHRA surveillance was 101 patients. Using the mean (range) per patient cost for surveillance, this represented a screening cost of £18,041 (range £14,924–£26,008) to avoid missing 1 case of asymptomatic ARMD which may need revision surgery in the future.

## Discussion

The effects of MoM bearings continue to be a source of worldwide concern. Increasingly intensive surveillance is recommended by national regulatory authorities in all MoMHA patients.^11^ This is on the basis that identifying problems early in asymptomatic patients will lead to timely investigation and further surgery if needed, which should improve outcomes.^27^ However the clinical evidence supporting the more intensive surveillance is questionable, especially in hip resurfacing patients with non-recalled implants,^13^ with data supporting the contrary that asymptomatic patients with hip resurfacings who have normal investigations have little change in blood metal ions and cross-sectional imaging when investigations are repeated within the first decade.^28–32^ It is understood that such regular surveillance in a large patient population is costly;^10^ however, we are unaware of any study examining the detailed costs for a patient recall which also considers the subsequent results of each investigation.

Implementing MHRA surveillance for “at-risk” BHR patients at our institution was extremely costly, both financially and logistically. At a time when recommendations are being made to reduce follow-up regularity for patients undergoing non-MoMHA,^33,34^ steps have been repeatedly taken to increase MoMHA surveillance.^5,11,15^ To review and investigate all eligible patients once was almost £106,000 (mean of £179 per patient), with the most expensive costs being imaging and advanced nurse practitioner assistance with clinics and administration. Our institution receives £67 per outpatient clinic appointment from the commissioner contract. Given there has been no contribution to follow-up and investigation costs from implant manufacturers, it is clear that all centres undertaking this and similar MHRA patient recalls will be running at a substantial financial deficit. Proponents of widespread surveillance for MoMHAs will suggest these excessive costs are justified as prophylaxis against litigation costs for missed cases of ARMD. However, this substantial financial deficit will only increase with time given the more intensive annual surveillance recommended for all MoMHA patients since 2017,^11,13^ the huge number of implants that remain *in situ* (39,104 MoM hip resurfacings have been implanted in the UK, with most still *in situ*), and the BHR representing the most commonly used resurfacing design worldwide.^14,35^ To further compound this issue, many MoMHAs were implanted in the private sector with the follow-up burden for these patients almost exclusively being covered by the National Health Service in the UK.^13^ These substantial financial and resource implications both now and in the future are concerning, whilst we face a time of increasingly depleted healthcare resources and budgets.

The cost of surveillance needs to be balanced against other factors including the severity, natural history, and treatment available for the condition potentially being identified earlier by performing surveillance. When little was known about ARMD, it was not clear how patients should be investigated or even treated, with poor outcomes reported after ARMD revision surgery.^27,36^ However this was a decade ago, and increasing awareness of investigating and treating ARMD have now led to improved outcomes for these patients which are comparable to revisions performed for non-MoMHAs.^13,37,38^ Therefore the implications of missing asymptomatic ARMD can no longer be considered to be as serious as missing certain cancers which could be detected from routine screening, and it is possible that the increasing surveillance in MoMHA patients is being driven by continued medicolegal and media pressures.^39,40^ Furthermore many asymptomatic ARMD patients eventually develop symptoms^41^ so would present, albeit a little later, when they become symptomatic, with recent evidence highlighting that patient self-referral followed by general practitioner referral are the commonest routes back to orthopaedic review.^34^ The risk of asymptomatic ARMD was low (1%) in our study, and we performed no revisions in asymptomatic patients. Given the above considerations, an NNT of 101 to avoid missing 1 case of asymptomatic ARMD is high. This NNT equates to £18,041 of surveillance to avoid missing 1 case of asymptomatic ARMD, which may or may not require future revision surgery. The cost of performing revision hip surgery for aseptic reasons is approximately £12,000,^42^ which is 33% cheaper than the cost of performing 1 round of surveillance in 101 patients, although in reality hospitals will receive much less reimbursement given tariffs for revision surgery have been cut by almost £3000.^43^ Therefore we consider the 2015 MHRA surveillance not to be cost efficient at our centre with the BHR implant, and have understandable concerns about the increasingly intensive surveillance proposed.^5,11,15^ However, it is important that all MoMHA patients are made aware of a clear route they can use to self-refer back to clinic in a timely manner if they do develop symptoms.

This work has primarily focused on the cost implications of the MHRA advice for MoMHA patients. However, an additional factor to consider is the diversion of valuable resources within our unit, which can be extrapolated to the whole healthcare system. Advanced Nurse Practitioners play a vital and varied role within Arthroplasty departments. This study includes approximately 68 hours of Advanced Nurse Practitioner time per month at our institution to manage MoMHA patients that have been recalled. If this could be reduced by half, 2 additional Advanced Nurse Practitioner clinics could be provided per week for other patients who may have significant clinical symptoms and need clinical review.

### Limitations

We acknowledge geographical variations in cost exist for each aspect of follow-up, which may mean the overall institutional costs for undertaking such a recall would vary between different centres and other countries that follow the MHRA guidance. The costs presented are likely an underestimate as we did not use cross-sectional imaging in all patients as described, and we have not incorporated the costs of revision surgery into the model given the threshold for revision is variable.^37^ As we adopted a selective policy for cross-sectional imaging, as per the advice at the time for asymptomatic stemmed THR patients,^5^ we may have missed some asymptomatic cases of ARMD on imaging which would decrease the NNT results presented. However, it should be noted that during our patient surveillance, the MHRA modified their recommendations in 2017 to perform selective cross-sectional imaging in asymptomatic hip resurfacing patients, which is in-line with what we had decided at our centre. It is acknowledged that a number of patients were untraceable or elected to be monitored elsewhere, which may introduce selection bias regarding the cohort presented as some of these patients may have asymptomatic ARMD. However, this is the reality of a mass patient recall, and our work highlights the logistical issues of following up many relatively young (mean age of 50 years at surgery) and active patients, most of which have no symptoms.

Our musculoskeletal radiologists are experienced with diagnosing ARMD in MoMHA patients, so abnormalities may not be detected on imaging with such accuracy at other centres without such radiological expertise. Our results are not generalisable to stemmed MoMHA designs, which are known to have higher failure rates than hip resurfacing, and may not be applicable to other MoM hip resurfacing implants that have an inferior outcome compared with the BHR.^14^ Finally, our centre has achieved good results with the BHR, with a large number of cases performed by 1 expert hip resurfacing surgeon,^22,23^ so the findings may not apply to centres with less experience with hip resurfacing where the rates of failure and/or ARMD may be higher with this implant.

## Conclusion

Implementing MHRA surveillance for “at-risk” BHR patients was extremely costly, both financially and logistically. As the risk of asymptomatic ARMD was low with the BHR (1%), our data suggests the 2015 MHRA surveillance is not cost efficient at our institution for asymptomatic BHR patients. This raises concerns about the increasingly intensive surveillance recommended in the 2017 MHRA guidance for metal-on-metal hip patients. We also have concerns that too much valuable Advanced Nurse Practitioner time is being used to monitor a specific group of patients very intensely at a time when significant waiting lists exist for other patients. Surveillance for MoMHA patients is important, however we feel the current guidelines should be reviewed for asymptomatic patients with well-performing hip resurfacing implants in light of the substantial costs presented here.
